# COVID-free surgical pathways for treating patients with acute calculous cholecystitis: a retrospective comparative study

**DOI:** 10.3389/fsurg.2024.1393948

**Published:** 2024-04-08

**Authors:** Pietro Giacopelli, Alessandra Cristaudi, Pietro Majno, Raffaello Roesel, Fabiano Iaquinandi, Francesco Mongelli

**Affiliations:** ^1^ Department of Surgery, Ospedale Regionale di Bellinzona e Valli, EOC, Bellinzona, Switzerland; ^2^ Department of Surgery, Ospedale Regionale di Lugano, EOC, Lugano, Switzerland; ^3^ Faculty of Biomedical Science, Università della Svizzera Italiana, Lugano, Switzerland

**Keywords:** cholecystitis, COVID, cholecystectomy, SARS-Cov, emergency surgery, pandemic

## Abstract

**Introduction:**

During the Sars-Cov-2 crisis, some of the resources committed to emergency surgery services were transiently reallocated to the care of patients with severe COVID-19, preserving immediate treatment of mostly non-deferrable conditions. Moreover, the fear of contracting infections or hindering the treatment of critical COVID-19 patients has caused many individuals to defer seeking emergency care. This situation has then possibly modified the standard of care of some common surgical conditions and the relative outcomes. Our aims was to highlight any difference in surgical outcomes in patients treated for acute cholecystitis before and during the COVID-19 outbreak.

**Method:**

This is a retrospective study on a prospectively collected database that included all consecutive patients treated for acute cholecystitis from March 2019 to February 2021 at the Lugano Regional Hospital, a COVID-free hospital for general surgery patients. Patients were divided into pre-and post-COVID-19 outbreak groups. We collected thorough clinical characteristics and intra-and postoperative outcomes.

**Results:**

We included 124 patients, of which 60 and 64 were operated on before and after the COVID-19 outbreak respectively. The two groups resulted similar in terms of patients' clinical characteristics (age, gender, body mass index, ASA score, and comorbidities). Patients in the post-outbreak period were admitted to the hospital 0.7 days later than patients in the pre-outbreak period (3.8 ± 6.0 days vs. 3.1 ± 4.1 days, *p* = 0.453). Operative time, recovery room time, complications, and reoperations resulted similar between groups. More patients in the post-outbreak period received postoperative antibiotic therapy (63.3% vs. 37.5%, *p* = 0.004) and for a longer time (6.9 ± 5.1 days vs. 4.5 ± 3.9 days, *p* = 0.020). No significant histopathological difference was found in operatory specimens.

**Discussion:**

Despite more frequent antibiotic therapy that suggests eventually worse inflammatory local status, our results showed similar outcomes for patients treated for acute cholecystitis before and during the COVID-19 pandemic. The local COVID management, reallocating resources, and keeping COVID-free hospitals was key to offering patients a high standard of treatment.

## 1 Introduction

The SARS-Cov-2 outbreak in 2019 challenged and overwhelmed health systems worldwide. At the beginning of the pandemic phase, the need to concentrate human and material resources in the care of critical COVID-19 patients is likely to have had an impact on the treatment of patients affected by common emergency surgical diseases (1). Limited access to the emergency departments has been also described as a consequence of patients' perceived fear and uncertainty (2–4). Moreover, the international recommendations in the early phase of the pandemic stressed the concept of avoiding surgery whenever possible in favour of conservative treatments (5). Acute cholecystitis is one of the most frequent acute conditions causing hospitalization and intervention (6), according to current guidelines, the standard of care for these patients should be a surgical intervention in absence of major contraindications (7, 8). Our hypothesis is that the COVID-19 outbreak may significantly affect the management of patients with acute cholecystitis with significant impact on short term outcomes. While the literature on the effects of COVID on oncological diseases is substantial (9–12), few studies have been conducted on this topic.

## 2 Materials and methods

For this is a retrospective study of a prospectively collected database of patients treated for acute cholecystitis, defined as acute inflammatory disease of the gallbladder presenting diagnostic criteria according to the Tokyo guidelines A non-objection letter was sent to all patients involved and the study was approved by the ethics committee (2021-01359 CE3908). We included all consecutive patients treated for acute cholecystitis at the Lugano Regional Hospital from March 2019 to February 2021. Our hospital was a COVID-free one, leaving surgical pathways free for urgent patients and redirecting COVID patients to a dedicated hospital in our region with both medical and surgical facilities. Data was extracted from medical records, then collected in a database and analyzed. Patients who underwent urgent cholecystectomy were treated according to the standard of care (13). We collected the following data from the patients' records: age, sex, indication for surgery, type of surgery, intra- and postoperative complications [according to the Clavien-Dindo classification (14)], operative time, presence of drainage, length of hospital stay, re-operation in the first 30 days, duration of postoperative antibiotic therapy, readmission rate. Patients were divided into pre- and post-COVID-19 outbreak groups. The primary endpoint was the complication rate between the two groups. Secondary outcomes included operative time, presence of drainage, length of hospital stay, re-operation in the first 30 days, duration of post-operative antibiotic therapy and readmission rate. Descriptive statistics were presented as absolute numbers and percentages for categorical variables and mean ± standard deviation (SD) for continuous variables. The comparison of dichotomous values was performed with the chi-squared test, while continuous variables were compared with the Student *t*-test. A *p*-value < 0.05 will be considered statistically significant. Statistical analysis will be performed on MedCalc® Statistical Software version 19.6 (MedCalc Software Ltd, Ostend, Belgium; https://www.medcalc.org; 2020).

## 3 Results

Over a 2-year period, 124 consecutive patients underwent cholecystectomy for acute cholecystitis at our institution. They were screened for eligibility and included in the present study. Sixty patients underwent surgery before and 64 after the COVID outbreak. The two study populations were similar in terms of demographic and clinical characteristics (Table 1). Specifically, the mean age was 61 ± 17 years in the pre-COVID period and 63 ± 14 years in the post-COVID period, and the sex distribution of patients was also similar in both groups. The two groups were also similar in terms of comorbidities such as cardiac, pulmonary, neoplastic, renal disease, diabetes and immunosuppression.

**Table 1:** Patient demographics and preoperative clinical characteristics.

	COVID-19-outbreak period *n* = 60	Pre-COVID-19 period *n* = 64	*p*
Age, years (SD)	61.3 (17.2)	63.3 (14.1)	0.484
Female gender, *n* (%)	21 (35.0)	27 (42.2)	0.413
BMI, kg/m^2^ (SD)	27.5 (5.2)	27.0 (4.1)	0.557
ASA score, *n* (%)			
I	26 (43.3)	27 (42.2)	0.802
II	18 (30.0)	20 (31.2)	
III	16 (26.7)	16 (25.0)	
IV	0	1 (1.6)	
Comorbidities, *n* (%)			
Cardiac disease/hypertension	18 (30.0)	26 (40.6)	0.218
Pulmonary disease	3 (5.0)	8 (12.5)	0.144
Diabetes type II	5 (8.3)	7 (10.9)	0.625
Tumor	1 (1.7)	3 (4.7)	0.343
Immunosuppression	1 (1.7)	0	0.302
Renal disease	3 (5.0)	6 (9.4)	0.350
Parameters upon ED arrival			
Body temperature, °C (SD)	36.8 (0.7)	36.9 (0.6)	0.665
Cardiac frequency, bpm (SD)	79 (13)	80 (14)	0.816
Tachypnea, *n* (%)	2 (3.3)	1 (1.6)	0.523
White blood cells, 10^9^/L (SD)	12.2 (5.1)	12.4 (4.5)	0.809
C-reactive protein, mg/L (SD)	93 (104)	122 (148)	0.206
Bilirubin, μmol/L (SD)	14 (21)	10 (14)	0.165
Time symptoms to surgery, days (SD)	3.8 (6.0)	3.1 (4.1)	0.453

ASA, American Society of Anesthesiology; BMI, body mass index; Bpm, beats per minute; ED, emergency department. Values are presented as mean with standard deviation (SD) or absolute number with percentage in parentheses.

We observed a significant difference between groups in both frequency and duration of post-operative antibiotic administration. In the group of patients operated during the COVID-19 crisis 38 (59.4%)patients received antibiotics, while before the outbreak it was administered in 24 (40.0%) patients only (*p* = 0.004). The antibiotics were administered for 6.9 ± 5.1 days vs. 4.5 ± 3.9 days in the post- and pre-outbreak period (*p* = 0.020). No difference was observed regarding intraoperative outcomes as operative time (104 ± 43 min vs. 115 ± 54 min, *p* = 0.195), preoperative antibiotic use (31 vs. 25 cases, *p* = 0.160), intraoperative complications (3 vs. 1 case, *p* = 0.281) and drain placement (4 vs. 4 cases, *p* = 0.925). No difference was also observed in length of stay (4.9 ± 3.6 days vs. 4.9 ± 4.8 days, *p* = 0.974) and postoperative complication rates (4 vs. 5 cases, *p* = 0.807). Details are shown in Table 2.

**Table 2:** Peri- and post-operative outcomes.

	COVID-19-outbreak period *n* = 60	Pre-COVID-19 period *n* = 64	*p*
Cholecystectomy, *n* (%)	60 (100)	64 (100)	1.000
Associated procedures			
Adhesiolysis, *n* (%)	2 (3.3)	0	0.113
Intraoperative ERCP, *n* (%)	4 (6.7)	1 (1.6)	
Operative time, min (SD)	104 (43)	115 (54)	0.195
Total operatory room time, min (SD)	179 (54)	168 (67)	0.317
Recovery room time, min (SD)	56 (43)	64 (33)	0.236
Preoperative antibiotics, *n* (%)	31 (51.7)	25 (39.1)	0.160
Intraoperative complications, *n* (%)	3 (5.0)	1 (1.6)	0.281
Drainage placement, *n* (%)	4 (6.7)	4 (6.2)	0.925
Hospital stay, days (SD)	4.9 (3.6)	4.9 (4.8)	0.974
Postoperative antibiotics, *n* (%)	38 (63.3)	24 (37.5)	**0** **.** **004**
Antibiotics duration, days (SD)	6.9 (5.1)	4.5 (3.9)	**0** **.** **020**
Postoperative complication, *n* (%)	4 (6.7)	5 (7.8)	0.807
Clavien-Dindo grade, *n* (%)			
I	3 (5.0)	2 (3.1)	0.386
II	0	1 (1.6)	
III	0	2 (3.1)	
IV	1 (1.7)	0	
Reoperations, *n* (%)	1 (1.7)	2 (3.1)	0.599

ERCP, endoscopic retrograde cholangiopancreatography. Values are presented as mean with standard deviation (SD) or absolute number with percentage in parentheses.

*P*-value <0.05 (in bold).

## 4 Discussion

Our study showed that the surgical treatment of patients with acute cholecystitis was not significantly affected by the COVID-19 crisis in a COVID-free hospital.

During the “lockdown period”, people were isolated for days without the possibility of being able to have close contact with relatives or neighbors. Out of fear of having contact with people who were positive for Coronavirus, many people who were experiencing problems avoided going to the doctor. We hypothesized that this could have led to late hospital admissions and more severe cholecystitis. Therefore, our study aimed at comparing the outcomes of patients undergoing emergency cholecystectomy at our institution before and after the COVID outbreak. We found that after COVID outbreak patients presented in the emergency department later than in the pre-SARS-CoV-2 phase although this difference was not statistically significant.

Regarding intraoperative results, timing and procedures performed were similar in both groups, with no differences for preoperative and postoperative complications. Other studies showed similar results confirming our findings in terms of intra- and postoperative complications, and length of hospital stay (15–17). In their study, Fabbri et al. (18) analyzed, among other interventions, cholecystectomies performed in the pre- and post-COVID period and found, as we did, no statistically significant differences in the degree of severity of presentation of acute cholecystitis, considered as conversion rate and gangrenous histotype.

However, our results differed from the findings of the CHOLECOVID study, an international, multicentre, observational comparative study of patients admitted to hospital with acute cholecystitis during the COVID-19 pandemic, that, as far as the authors know, is the largest conducted so far on this argument (19). The authors found out a significant shift in the severity of acute cholecystitis with more grade II [1,653 patients (30 per cent) pre-pandemic compared with 1,499 patients (35 per cent pandemic)] and more grade III disease [208 patients (3.8 per cent pre-pandemic) compared with 175 patients (4.1 per cent pandemic; *p*, 0.001)] and speculated that the shift in admission severity reflected delayed presentation. In our experience, we believe that the key to providing optimal surgical treatment and the reason why our results are different, was the institution of COVID-free surgical pathways. In addition, the segregation of COVID-positive patients and their subsequent transfer to facilities dedicated to their care allowed for a COVID-free hospital. This allowed the gold standard treatment to be offered to patients without the need for other, less invasive methods described by other centres (20, 21). This interpretation also explains the difference in results with the CHOLECOVID study: a multi-centre study that did not include only hospitals with a COVID-free pathway.

An interesting finding was the significant difference we found in the duration of antibiotic therapy administered during and after hospitalization. The physician's desire to avoid potential complications requiring readmission, with the consequent risk of exposing the patient to SARS-CoV-2 infection, or saturation of hospital resources may explain the use of antibiotics in the postoperative period. This may have resulted in case-specific treatment decisions and deviation from what may be normal EBM-guided clinical practice. Another possible explanation is that there was a difference in the severity of acute cholecystitis at the time of hospital admission, which is consistent with the literature.

We believe our study is meaningful as the creation of COVID-free surgical pathways did not delayed the assessment of patients with cholecystitis. Despite the fact that the data relate to the period 2019 and 2021, with a delay in the publication of the results mainly due to a later conception and design of the study rather than a difficulty in data collection, we believe that these results may still be relevant. In fact, they are applicable to any type of public health emergency that leads to a reallocation of hospital resources, as well as to a still current pandemic risk. Our results are generalizable to all hospital and regional healthcare facilities in case of new severe COVID variants. The possibility to reserve COVID-free hospital, if permitted by resources, was key in our experience and is consistent with the results of other studies conducted on surgical COVID-free surgical pathways (22, 23). Our study has several limitations. Firstly, the retrospective design and the limited number of patients. We could not measure the time spent waiting for an operating theatre to be available. As we know, during COVID the waiting time for operating theatres was increased due to reduced staffing and isolated patients who required longer preparation time with a reduced number of instruments. Perhaps this unstudied element could motivate the prolonged administration of antibiotics in the preoperative period, which ultimately led to different outcomes in the two groups.

## 5 Conclusions

Before and during the COVID-19 outbreak no significant difference in the treatment of acute cholecystitis was noted. The key to an optimal treatment was having COVID-free surgical pathways and hospitals.

## Data Availability

The raw data supporting the conclusions of this article will be made available by the authors, without undue reservation.
